# Extreme dipping blood pressure pattern is associated with increased mortality in hemorrhagic stroke patients: a retrospective cohort study

**DOI:** 10.1186/s12883-025-04342-x

**Published:** 2025-08-02

**Authors:** Feng Nong, Wei Zhu, Yan Jiang

**Affiliations:** 1 Department of Nursing, West China Hospital Sichuan University , West China School of Nursing, Sichuan University, Chengdu, China; 2 Evidence-based Nursing Center, West China Hospital, Sichuan University, Chengdu, China; 3 Sichuan Provincial Engineering Research Center of Medical Nursing Equipment and Materials, Chengdu, China

**Keywords:** Mortality, Circadian rhythm, Intensive care unit, Circadian blood pressure patterns, Hemorrhagic stroke, Blood pressure

## Abstract

**Background:**

Blood pressure management strategies in patients with hemorrhagic stroke remain ineffective in reducing mortality. The circadian blood pressure pattern has been shown to be associated with mortality in patients with non-hemorrhagic stroke, but few studies have explored this association in patients with hemorrhagic stroke. We aimed to investigate the relationship between circadian blood pressure pattern and mortality in patients with hemorrhagic stroke.

**Methods and results:**

Adult hemorrhagic stroke patients hospitalized in intensive care unit for more than 24 h in the Medical Information Mart for Intensive Care (MIMIC-IV) database were recruited for this retrospective cohort study. All patients were divided into the dipping group, the nondipping group, the reverse dipping group and the extreme dipping group. We used binary logistic regression analysis to explore the relationship between circadian blood pressure patterns and mortality of patients with hemorrhagic stroke. The overall cohort comprised 1040 patients. The patients in the extreme dipping group had higher mortality than other groups (57.1% versus 15.6%,17.0%, and 22.3%, respectively). After adjusting for covariates, the statistical analysis showed that the extreme dipping pattern was significantly associated with the mortality of hemorrhagic stroke patients in intensive care unit (odds ratio: 4.961[95%CI: 1.289–19.086]). Interaction analysis had no statistical significance in all results.

**Conclusions:**

The extreme dipping pattern may be an important risk factor for increased mortality in patients with hemorrhagic stroke.

## Introduction

Hemorrhagic stroke, which includes intracranial hemorrhage (ICH) and subarachnoid hemorrhage (SAH), is a severe and life-threatening disease that requires immediate medical attention. Although hemorrhagic stroke represents a smaller portion of all strokes, approximately 20%, it has a high likelihood of early mortality and long-term disability. The clinical outcomes of hemorrhagic stroke patients can be substantially improved by employing prompt and precise therapeutic interventions within the intensive care unit [1–4]. The management of blood pressure is an important aspect of improving the outcomes in hemorrhagic stroke patients [1, 5, 6]. Early and stable management of blood pressure was safe in hemorrhagic stroke patients, and helps to improve the functional outcome of hemorrhagic stroke patients [7]. In 2008, the INTERACT study, a randomized pilot trial involving 404 hemorrhagic stroke patients, found that early lowering of systolic blood pressure below 140 mm Hg seemed to reduce hematoma expansion but not mortality in these patients [8], while hematoma expansion, an appealing therapeutic target, affects 20% of patients within 24 h of the onset of acute intracerebral hemorrhage and is associated with death, disability, and early neurological deterioration [9, 10]. In 2013, the INTERACT2 study, a multicenter and international randomized controlled trial including 2,839 hemorrhagic stroke patients, found that lowering the systolic blood pressure below the same target within 1 h helped to improve the functional outcome but still not survival rate in hemorrhagic stroke patients [11]. In 2023, the INTERACT3 study, a stepped-wedge cluster and international randomized controlled trial including 7,036 hemorrhagic stroke patients, also found that managing patients’ glucose, temperature, anticoagulation, and systolic blood pressure below the same target led to similar favorable functional outcomes. However, those management strategies were still ineffective in reducing patients’ mortality rates, only managing to significantly reduce the 6-month mortality rate [12]. Other blood pressure management strategies, such as the ATACH study, also found that by lowering early systolic blood pressure more aggressively in patients with hemorrhagic stroke compared to the INTERACT studies, there was still no significant difference in mortality between the experimental and control groups [13]. In conclusion, clinical blood pressure management strategies can improve functional outcomes but are still ineffective in reducing mortality in hemorrhagic stroke patients and it can also be found that clinical blood pressure management is mainly performed by controlling the absolute value of systolic blood pressure rather than by circadian blood pressure patterns.

The circadian blood pressure pattern manifests as the variation of blood pressure within 24 h. Specifically, the blood pressure of a healthy individual is typically higher during periods of wakefulness and lower during sleep, showing a cyclical variation [14, 15]. The circadian blood pressure pattern has been shown to be associated not only with patient mortality but also with patient functional outcomes. Ding et al. performed a study involving 1,808 patients with acute ischemic stroke or transient ischemic attack found that the circadian blood pressure pattern may be a risk factor for patients’ functional outcome [16]. A cohort study performed by Mayer found that the circadian blood pressure pattern may increase the all-cause mortality of hemodialysis patients [17]. A prospective cohort study involving 588 patients with chronic kidney disease reported that the reverse dipping pattern was associated with higher total and cardiovascular mortality, renal events and cardiovascular events [18]. Some studies have investigated the association between circadian blood pressure patterns and the risk of stroke, often involving mixed populations of ischemic and hemorrhagic stroke patients [19, 20]. However, a few studies have specifically examined the relationship between circadian blood pressure patterns and clinical outcomes in patients with hemorrhagic stroke. Investigating the effect of circadian blood pressure patterns on mortality outcomes in patients with hemorrhagic stroke through a retrospective cohort study may provide a clinical intervention that could potentially improve mortality rates of hemorrhagic stroke patients.

Thus, this study aims to explore the relationship between the circadian blood pressure patterns and the mortality of hemorrhagic stroke patients, with the aim of enhancing early risk stratification of hemorrhagic stroke patients and exploring additional intervention perspectives.

## Methods

### Study design and population

This was a retrospective cohort study. The patients’ information of our study was obtained from the MIMIC-IV (version 2.2), which is a publicly accessible and comprehensive critical care database maintained by the Computational Physiology Laboratory at the Massachusetts Institute of Technology (MIT) [21]. The database contains detailed records of approximately 73,000 hospitalizations in the intensive care unit at Beth Israel Deaconess Medical Center over a ten-year period from 2008 to 2019. According to the Ninth Revision (ICD-9) and Tenth Revision (ICD-10) of International Classification of Diseases, hemorrhagic stroke patients were recruited for this study. Specifically, we selected the following ICD codes for intracranial hemorrhage and nontraumatic subarachnoid hemorrhage: ICD-9 code 430, 431, 4329, 7670, 77,210–77,214 and ICD-10 codes I60, I61, I62, I601-I609, I610-I619, I600, I6000-I6002, I6010-I6012, I6020-I6022, I629, I6030-I6032, I6050-I6052. Patients with the following conditions were excluded from this study: [1] less than 18 years of age; [2] intensive care unit stays less than 24 h; [3] multiple intensive care unit admissions; [4] daytime blood pressure checks less than 10 times; [5] nighttime blood pressure checks less than 7 times.

### Data extraction

In order to obtain the data, Wei Zhu, one of the authors, completed the training course on protecting human research participant provided by the National Institutes of Health (NIH) and obtained certification from the Collaborative Institutional Training Initiative (Record ID: 62,749,768).Using Navicat Premium software (version 17) for data retrieval, the software extracted information from seven different categories using structured query language (SQL): clinical characteristics, sociodemographic variables, laboratory parameters, comorbidities, vital signs, records of medication and surgical procedure. Sociodemographic variables included sex, age and weight. Records of medication and surgical procedure included use of antihypertensive therapy and brain surgery intervention.

### Circadian blood pressure patterns

In this cohort study, the primary exposure factor we investigated was the circadian blood pressure patterns observed during the first 24 h after ICU admission in patients with acute intracerebral hemorrhage. We noted that under normal physiological circumstances, blood pressure demonstrates a pronounced circadian rhythm, where nighttime systolic blood pressure is characterized by a notable decrease of 10–20% when compared to the systolic blood pressure levels measured during the daytime. This decline in nighttime systolic blood pressure serves as a significant indicator that reflects the circadian rhythm governing blood pressure variability throughout the day. The nocturnal systolic blood pressure decrease ratio, calculated as [mean daytime systolic blood pressure] – [mean nighttime systolic blood pressure]/mean daytime systolic blood pressure × 100%, allows for the categorization of circadian blood pressure patterns into four groups: dipping group (10–20%), nondipping group (0–10%), reverse dipping group (< 0%), and extreme dipping group (> 20%). In this study, blood pressure-related parameters were derived from non-invasive blood pressure measurements collected near each hourly time point in the MIMIC-IV database. To ensure consistency in circadian rhythm assessment, only blood pressure data from the first 24 h after ICU admission were included in the analysis. The mean daytime systolic blood pressure was calculated by averaging systolic blood pressure values recorded between 8:00 AM and 9:59 PM, while the mean nighttime systolic blood pressure was calculated using values recorded between 10:00 PM and 7:59 AM.

### Outcomes

In our study, the primary outcome measure was hospital mortality of hemorrhagic stroke patients.

### Statistical analysis

In this study, the data of continuous variables was tested for normality by using the Kolmogorov-Smirnov test or Shapiro-Wilk test, depending on the sample size of groups. The continuous variable data that met normality and homogeneity of variance was expressed as mean ± standard deviation and tested for significance by using the T-test. The continuous variable data that do not fit a normal distribution and discrete variable data were expressed as median (interquartile range) and tested for significance by using the Mann–Whitney test. Categorical variables data was expressed as frequency and percentage and tested for significance by using the Chi-square test. The univariate analyses above had two-side alpha levels of 5%. We use multivariate logistic regression analysis to explore the association between circadian blood pressure patterns and the mortality of study population. Variables were selected according to their statistical significance in the univariate analysis as well as clinical significance into the multivariate logistic regression model. The purpose of including covariates was only to adjust the regression analysis and control for confounding, not to explore their relationship with the outcome of this study, so we did not transform the continuous variable data into ordered categorical variable data or by a factor of 10 in regression analysis to better explain their clinical significance. All statistical analyses were conducted using the statistical software package IBM SPSS Statistics for Windows, version 27.0 (IBM Corp., Armonk, N.Y., USA).

## Results

### Patient selection, characteristics, and outcomes

**Fig. 1:**
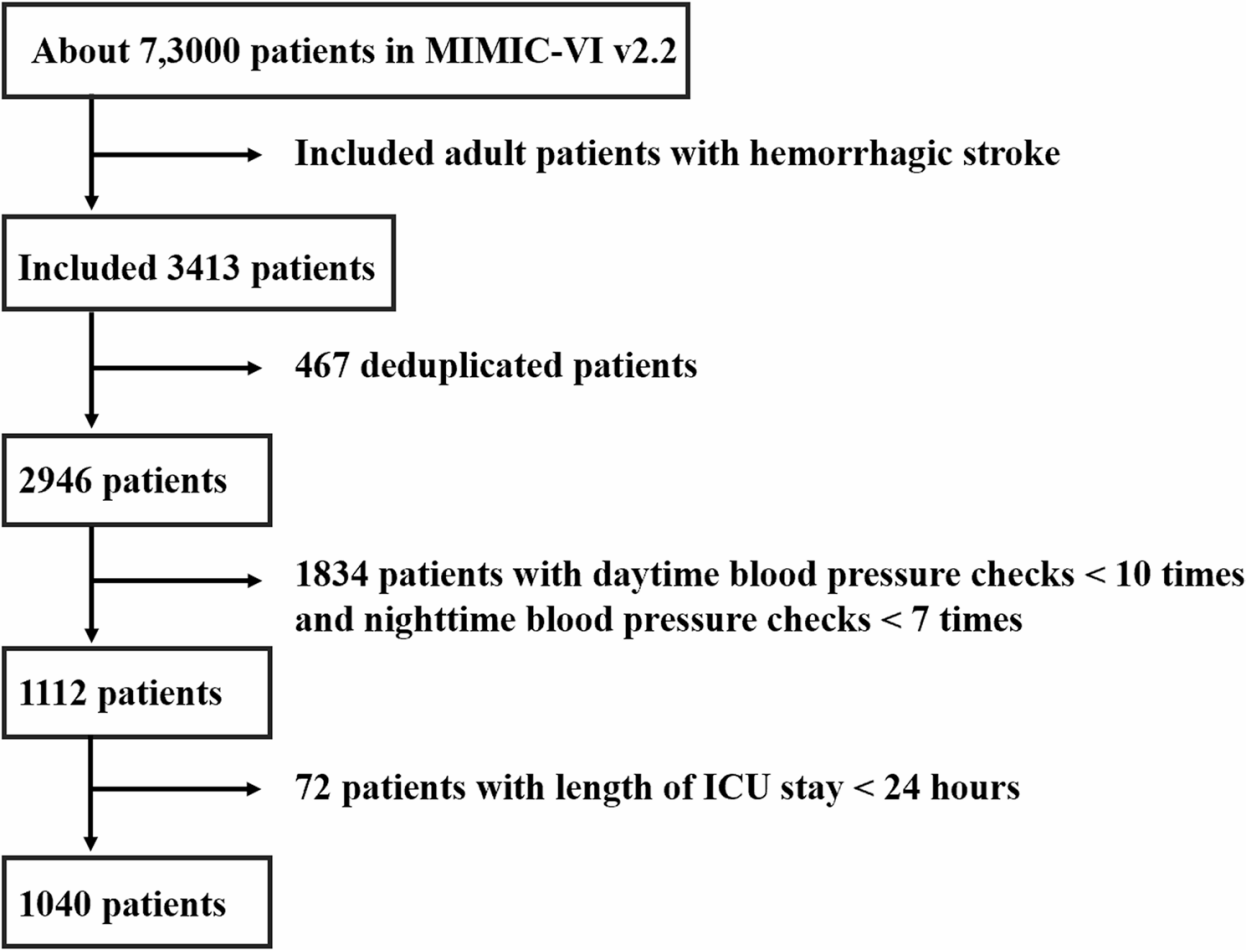
Flowchart for patient inclusion and exclusion. Callouts: 1040 patients were included in this study. MIMIC-IV, Medical Information Mart for Intensive Care; ICU, intensive care unit

All data obtained from the MIMIC-IV with a total of 7,3000 patients. 3413 patients were included after we searched the database for the first intensive care unit admission and ≥ 18 years. We excluded 467 patients with deduplicated data, 1834 patients with daytime blood pressure checks<10 times and nighttime blood pressure checks<7 times, 72 patients with length of ICU stay<24 h. Finally, 1040 patients were included in this study (Fig. 1). The average age of the 1,040 patients was 69.1 ± 15.33 years, and 48.3% were male. Mean daytime systolic blood pressure averaged 130.2 ± 14.90 mmHg; Mean nighttime systolic blood pressure averaged 127.2 ± 15.58 mmHg. The extreme dipping group had 14 patients. Please see the details of all values in Table 1.

**Table 1:** Characteristics of included patients

Item	Dipping(*n* = 128)	Nondipping (*n* = 513)	Reverse dipping (*n* = 385)	Extreme dipping (*n* = 14)	H/χ2	*p*
Stroke type (%)					6.42	0.092
ICH	93 (72.7%)	416 (81.1%)	307 (79.7%)	9 (64.3%)		
SAH	35 (27.3%)	97 (18.9%)	78 (20.3%)	5 (35.7%)		
LOS (SD)	5.5 (7.47)	4.6 (5.14)	5.5 (6.26)	3.7 (2.68)	1.79	0.617
Age (SD)	68.3 (14.49)	68.2 (15.70)	70.4 (15.15)	71.4 (12.31)	5.20	0.158
Gender (%)					1.13	0.770
Male	66 (51.6%)	240 (46.8%)	189 (49.1%)	7 (50.0%)		
Female	62 (48.4%)	273 (53.2%)	196 (50.9%)	7 (50.0%)		
Race (%)					19.19	0.084
White	80 (62.5%)	319 (62.2%)	256 (66.5%)	8 (57.1%)		
Black	8 (6.2%)	44 (8.6%)	31 (8.1%)	1 (7.1%)		
Asian	3 (2.3%)	24 (4.7%)	10 (2.6%)	2 (14.3%)		
HISPANIC/LATINO	4 (3.1%)	26 (5.1%)	6 (1.6%)	0 (0.0%)		
Unknown/Other	33 (25.8%)	100 (19.5%)	82 (21.3%)	3 (21.4%)		
GCS (IQR)	14.0 (11.8–15.0)	14.0 (11.0–15.0)	14.0 (11.0–15.0)	14.0 (12.5–15.0)	1.55	0.670
Blood pressure (SD)						
Nighttime SBP	117.1(11.92)	125.0(13.57)	134.1(16.16)	108.7(9.00)	164.54	< 0.01
Daytime SBP	135.3(13.83)	131.2(14.26)	126.7(15.30)	141.6(11.75)	49.37	< 0.01
24 h-SBP CV	12.67% (3.32%)	9.72% (2.86%)	10.69% (3.92%)	21.37% (7.16%)	116.30	< 0.01
Antihypertensive therapy (%)	64 (50.0%)	284 (55.4%)	207 (53.8%)	10 (71.4%)	2.89	0.410
Surgical intervention (%)	3 (2.3%)	16 (3.1%)	16 (4.2%)	0 (0.0%)	1.73	0.629
Mortality (%)					18.05	< 0.01
Death	20 (15.6%)	87 (17.0%)	86 (22.3%)	8 (57.1%)		
Survival	108 (84.4%)	426 (83.0%)	299 (77.7%)	6 (42.9%)		

*ICH* Intracerebral hemorrhage, *SAH* Subarachnoid hemorrhage, *LOS* Length of stay, *SD* Standard deviation, *GCS* Indicates Glasgow coma scale, *SBP* Systolic blood pressure, *CV* Coefficient of variation

#### Association between circadian blood pressure pattern and mortality

The hemorrhagic stroke patients in the extreme dipping group had higher mortality than those in the other three circadian blood pressure pattern groups(57.1% versus 15.6%、17.0%、22.3%).To verify the outcome, multivariate logistic regression was used by adjusting all covariates, including age, race, Glasgow Coma Scale score [22], mean daytime systolic blood pressure, mean nighttime systolic blood pressure, antihypertensive therapy, surgical procedure, the coefficient variation of 24-hour systolic blood pressure (24 h-SBP CV) and circadian blood pressure patterns. The results were similar to those of univariate regression analysis (hospital mortality: OR: 4.961[95%CI: 1.289–19.086] (Table 2).

**Table 2:** The risk factors of mortality of hemorrhagic stroke patients in logistic regression analysis

Variables	OR	95%CI	*p*
Circadian patterns			
dipping			0.120
nondipping	1.251	0.631-2.482	0.521
reverse dipping	1.17	0.439-3.121	0.754
extreme dipping	4.961	1.289-19.086	0.020
Age	1.024	1.012-1.036	<0.01
Race	1.153	1.051-1.264	<0.01
GCS	0.918	0.874-0.965	<0.01
Nighttime SBP	1.014	0.984-1.044	0.387
Daytime SBP	0.980	0.950-1.010	0.187
24h-SBP CV	1.095	1.047-1.145	<0.01
Antihypertensive therapy	0.922	0.644-1.280	0.682
Surgical intervention	3.695	1.811-7.541	<0.01

*24h-SBP CV* Coefficient of variation of 24-hour systolic blood pressure, *GCS* Glasgow coma scale, *OR* Odds ratio, *SE* Standard error, *SBP* Systolic blood pressure

## Discussion

Our findings revealed a significant association between the extreme dipping pattern and higher mortality in hemorrhagic stroke patients. Specifically, hemorrhagic stroke patients with extreme dipping pattern exhibited a mortality rate of 57.1%, compared to 15.6%, 17.0%, and 22.3% in the dipping, nondipping, and reverse dipping pattern, respectively. After adjusting for covariates, the extreme dipping pattern was significantly associated with increased mortality compared to the dipping pattern (OR: 4.961; 95% CI: 1.289–19.086). Additionally, age (OR: 1.024; 95% CI: 1.012–1.036), race (OR: 1.153; 95% CI: 1.051–1.264), Glasgow Coma Scale (GCS) score (OR: 0.918; 95% CI: 0.874–0.965), surgical intervention (OR: 3.695; 95% CI: 1.811–7.541), and 24-hour systolic blood pressure coefficient of variation (24 h-SBP CV; OR: 1.095; 95% CI: 1.047–1.145) were also independently associated with mortality in the regression model.

In addition to circadian blood pressure patterns, several other variables in our regression model—including age, Glasgow Coma Scale score, race, surgical intervention and 24-hour systolic blood pressure variability—were also significantly associated with mortality. Consistent with prior research linking these factors to adverse outcomes after stroke, age has been repeatedly identified as an independent risk factor for poor outcomes in patients with intracerebral hemorrhage [23–25], possibly attributable to larger hematoma volumes and reduced physiological reserve in older individuals. Similarly, lower admission Glasgow Coma Scale scores are established strong predictors of early mortality [26]. Racial disparities may also constitute a contributory factor, as differences in stroke incidence and prognosis across racial groups have been reported in the literature [27]. Elevated 24-hour systolic blood pressure variability has been independently associated with higher mortality risk [28, 29], underscoring the importance of blood pressure stability beyond absolute values. Furthermore, prior studies have also indicated that timely surgical intervention—particularly minimally invasive approaches—may improve outcomes in selected patients with intracerebral hemorrhage [3].

Circadian rhythms, manifesting in a 24-hour cycle, govern various biological processes such as blood pressure, sleep-wake cycles, metabolism, and body temperature. These periodic changes are regulated by the central clock located in the supraoptic nucleus of the hypothalamus and peripheral clocks situated in various parts of the body. Through signals from light and diet, these clocks are synchronized with daily time, which in turn affects the regulation of a wide range of physiological activities with important implications for health. Circadian rhythms influence blood pressure variability in humans, a phenomenon that has also been observed in mice. Blood pressure, as the product of cardiac output and total peripheral resistance, is regulated by several systems including the sympathetic, central nervous, renal, cardiac, vascular and immune systems [15].

Similar to the results of the present study, a substantial body of research has consistently demonstrated that abnormal circadian rhythm of blood pressure is strongly linked to negative health outcomes, including an increased risk of cardiovascular events and mortality. Fagard et al. conducted a meta-analysis of individual data from 3468 patients in 4 European prospective studies and showed that daytime and nighttime systolic blood pressure were associated with all-cause and cardiovascular mortality [30]. Different dipping blood pressure patterns were associated with impaired left atrial phasic function by comparing the results of comprehensive echocardiographic examination of 256 untreated hypertensive patients [31]. Palatini et al. performed an analysis enrolled 10,868 participants in 8 prospective studies founded the association between extreme dippers and adverse outcomes in elder patients over 70 years of age [32]. The Ohasama study, a long-term prospective cohort study following 1430 Japanese subjects, observed that extreme dipping pattern had a higher risk of brain hemorrhage compared to the dipping pattern [33]. However, a few studies have reported the impact and the prognostic value of different blood pressure dipping patterns in hemorrhagic stroke patients.

The effect of extreme dipping patterns on patients remains controversial, with mixed results in various previous studies. A study comprising 59,124 Spanish patients showed that extreme dipping pattern did not increase the risk of mortality of patients with cardiovascular diseases [34]. Even in a meta-analysis of four European studies, the opposite conclusion to the present study was reached that extreme dipping pattern was associated with a reduced risk of all-cause mortality in hypertensive patients. The differences between the findings of these studies may be attributed to the heterogeneity of study subjects. Previous studies have indicated that dramatic fluctuations in blood pressure among patients with hemorrhagic stroke can impair cerebral autoregulation and this impairment leads to increased hydrostatic and oncotic pressure gradients in the region surrounding the hematoma, thereby exacerbating the severity of cerebral edema [35, 36].

In previous clinical studies of blood pressure management strategies in patients with hemorrhagic stroke, including a series of INTERACT and ACATH studies [8, 11–13], the benefit of early, rapid and steady reductions in patients’ systolic blood pressure on patients’ functional outcomes has been demonstrated. However, no such benefit was observed in terms of patient mortality, which may be related to the lack of attention to the different circadian blood pressure patterns of hemorrhagic stroke patients in the studies mentioned above. We therefore conducted a retrospective cohort study that included 1,040 hemorrhagic stroke patients, hoping to provide an intervention to improve outcomes in patients with hemorrhagic stroke by exploring the causal relationship between circadian blood pressure patterns and mortality. It is recommended that the future clinical randomized controlled trials of blood pressure management strategies in hemorrhagic stroke patients should not only focus on controlling the absolute value of systolic blood pressure but also incorporate patients’ varying circadian blood pressure patterns into the trial design.

This study still has some limitations that should be considered. First, the primary data source of this study was the MIMIC-IV database, which is a single-center dataset; therefore, Berkson bias is inevitable. Second, several important covariates associated with patients with cerebral hemorrhage—such as hematoma volume, body mass index (BMI), and the modified Rankin Scale (mRS)—were not available in the database and thus were not included in our analysis. Our final logistic regression analysis only included age, race, Glasgow Coma Scale (GCS) score, mean daytime systolic blood pressure, mean nighttime systolic blood pressure, use of antihypertensive therapy, surgical intervention, the coefficient variation of 24-hour systolic blood pressure (24 h-SBP CV) and circadian blood pressure patterns. Therefore, the limited number of covariates included in the regression model may have compromised the reliability of the study findings. Third, this study is a retrospective cohort study that investigated only the association between circadian blood pressure pattern and mortality rather than establishing a causal relationship. Fourth, the small sample size of this study led to the extreme dipping pattern group only involving 14 patients, which weakened the confidence in the validity of the study conclusions. We still need to conduct a multi-center and well-designed prospective study with adequate sample size and covariates to further confirm the relationship between circadian blood pressure pattern and the mortality of hemorrhagic stroke patients.

## Conclusions

The extreme dipping pattern may be an important risk factor for hemorrhagic stroke patients.

## Data Availability

The datasets used and/or analysed during the current study were obtained from the MIMIC-IV database and are available from the co-author on reasonable request.

## Abbreviations

MIMIC-IV: Medical Information Mart for Intensive Care
ICH: Intracranial hemorrhage
SAH: Subarachnoid hemorrhage
INTERACT: Intensive Blood Pressure Reduction in Acute Cerebral Hemorrhage Trial
ATACH: Antihypertensive Treatment of Acute Cerebral Hemorrhage
ICD: International Classification of Diseases
NIH: National Institutes of Health
SQL: Structured query language
AM: Ante meridiem
PM: Post meridiem
IBM: International Business Machines Corporation
SPSS: Statistical Package for the Social Sciences
Corp: Corporation
N.Y.: New York
USA: United States of America
SBP: Systolic blood pressure
CV: Coefficient of variation
GCS: Glasgow coma scale
SD: Standard deviation
OR: Odds ratio
CI: Confidence interval
ICU: Intensive care unit
